# [1*R*-(1α,2α,4α,5β,6α,7α)]-4-Benzoyl­oxymethyl-5,6-dihy­droxy-3,8-dioxa­tricyclo­[5.1.0.0^2,4^]octan-5-yl acetate (3-deacetyl­crotepoxide) from *Kaempferia rotunda* Val.

**DOI:** 10.1107/S1600536810042686

**Published:** 2010-10-30

**Authors:** Hasnah Mohd Sirat, Yau Sui Feng, Khalijah Awang, Seik Weng Ng

**Affiliations:** a Department of Chemistry, Universiti Teknologi Malaysia, 81310 Skudai, Malaysia; b Department of Chemistry, University of Malaya, 50603 Kuala Lumpur, Malaysia

## Abstract

The title compound, C_16_H_16_O_7_, isolated from *Kaempferia rotunda* rhizomes, features a six-membered cyclo­hexane ring that adopts a twisted-boat conformation owing to the presence of two adjacent epoxide attachments that lock in four of the six axial positions. The CH_3_CO_2_– and HO– substituents occupy equatorial positions. However, the bond angles at the ring carbon connected to the C_6_H_5_CO_2_CH_2_– substituent deviate signifcantly from the idealized tetra­hedral angles as the carbon atom is part of an epoxide ring.  In the crystal, the molecules are linked into chains by O—H⋯O hydrogen bonds.

## Related literature

For the isolation of the compound from *Kaempferia rotunda*, see: Pancharoen *et al.* (1996 ▶).
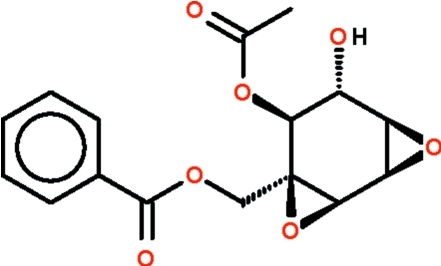


## Experimental

### 

#### Crystal data

C_16_H_16_O_7_
*M* *_r_* = 320.29
Orthorhombic, 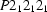

*a* = 5.7451 (7) Å
*b* = 7.1746 (9) Å
*c* = 35.708 (5) Å
*V* = 1471.9 (3) Å^3^
*Z* = 4
Mo *K*α radiation
μ = 0.12 mm^−1^
*T* = 100 K
0.35 × 0.05 × 0.05 mm

#### Data collection

Bruker SMART APEX diffractometer
14228 measured reflections
2011 independent reflections
1730 reflections with *I* > 2σ(*I*)
*R*_int_ = 0.073

#### Refinement

*R*[*F* ^2^ > 2σ(*F* ^2^)] = 0.038
*wR*(*F* ^2^) = 0.117
*S* = 1.12
2011 reflections
213 parameters
1 restraint
H atoms treated by a mixture of independent and constrained refinement
Δρ_max_ = 0.37 e Å^−3^
Δρ_min_ = −0.33 e Å^−3^

### 

Data collection: *APEX2* (Bruker, 2009 ▶); cell refinement: *SAINT* (Bruker, 2009 ▶); data reduction: *SAINT*; program(s) used to solve structure: *SHELXS97* (Sheldrick, 2008 ▶); program(s) used to refine structure: *SHELXL97* (Sheldrick, 2008 ▶); molecular graphics: *X-SEED* (Barbour, 2001 ▶); software used to prepare material for publication: *publCIF* (Westrip, 2010 ▶).

## Supplementary Material

Crystal structure: contains datablocks global, I. DOI: 10.1107/S1600536810042686/bt5386sup1.cif

Structure factors: contains datablocks I. DOI: 10.1107/S1600536810042686/bt5386Isup2.hkl

Additional supplementary materials:  crystallographic information; 3D view; checkCIF report

## Figures and Tables

**Table 1:** Hydrogen-bond geometry (Å, °)

*D*—H⋯*A*	*D*—H	H⋯*A*	*D*⋯*A*	*D*—H⋯*A*
O5—H5⋯O4^i^	0.84 (3)	2.05 (3)	2.887 (3)	172 (4)

Symmetry code: (i) 
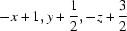
.

**Fig. 1.**
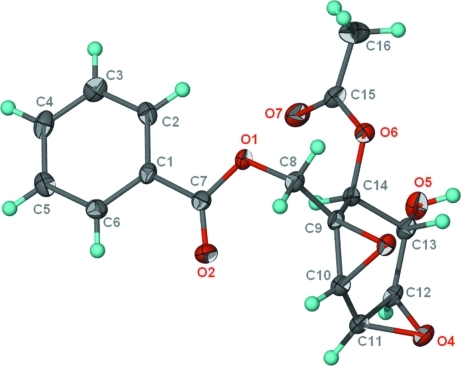
Anisotropic displacement ellipsoid plot (Barbour, 2001) of C16H16O7 at the 70% probability level; hydrogen atoms are drawn as spheres of arbitrary radius.

## supplementary crystallographic information

### Comment

*Kaempferia rotunda* is one of the four Malaysian *Kaempferia* of the Zingiberaceae family; among the constitutents isolated is 3-deacetylcrotepoxide (Scheme I), which was first reported by Pancharoen *et al.* (1996). 3-Deacetylcrotepoxide (Scheme I) features a six-membered cyclohexane ring that adopts a twisted boat conformation owing to the presence of two adjacent epoxide attachements that tie up four of the six axial positions. The CH_3_CO_2_– and HO– substituents occupy regular equatorial positions as each is connected to a methine carbon atom (Fig. 1). However, the bond angles at the ring carbon connected to the C_6_H_5_CO_2_CH_2_– substituent deviate signifcantly from the idealized tetrahedral angles as the carbon atom is part of an epoxide ring [112.4 (2), 117.9 (2), 120.3 (3) °].

### Experimental

*Kaempferia rotunda* rhizomes were purchased from a market in Kempas, Johor. The rhizomes were dried and then grounded. The grounded rhizomes were extracted with *n*-hexane (4.5 *L*), ethyl acetate (4.5 *L*) and methanol (4.5 *L*) in a soxhlet extractor for 16 h. The extracts were concentrated to give a dark brown semi-solid from the n-hexane extract (2.32 g), a dark brown oil from the ethyl acetate extract (6.80 g) and a dark brown viscous liquid from the methanol extract (15.27 g). The ethyl acetate extract (6.80 g) was fractionated by VLC (260 g, column size: 10 *x* 12 cm) by using petroleum ether, ether and ethyl acetate to afford four fractions, (0.02 g, 0.15 g, 2.70 g and 2.50 g. Evaporation of the solvent of the third fraction yielded 3-deacetylcrotepoxide (0.145 g, 2.13%) as colorless crystals.

The absolute configuration was assumed from that obtained from spectroscopic measurements (Pancharoen *et al.*, 1996).

### Refinement

Carbon-bound H-atoms were placed in calculated positions (C—H 0.95–0.99 Å) and were included in the refinement in the riding model approximation, with *U*(H) set to 1.2–15*U*(C).

The hydroxy H-atom was located in a difference Fourier map, and was refined isotropically with the O–H distance restrained to 0.84±0.01 Å.

1374 Friedel pairs were merged.

### Crystal data

**Table:**

C_16_H_16_O_7_	*F*(000) = 672
*M**_r_* = 320.29	*D*_x_ = 1.445 Mg m^−^^3^
Orthorhombic, *P*2_1_2_1_2_1_	Mo *K*α radiation, λ = 0.71073 Å
Hall symbol: P 2ac 2ab	Cell parameters from 1788 reflections
*a* = 5.7451 (7) Å	θ = 3.1–20.0°
*b* = 7.1746 (9) Å	µ = 0.12 mm^−^^1^
*c* = 35.708 (5) Å	*T* = 100 K
*V* = 1471.9 (3) Å^3^	Prism, colorless
*Z* = 4	0.35 × 0.05 × 0.05 mm

### Data collection

**Table:**

Bruker SMART APEX diffractometer	1730 reflections with *I* > 2σ(*I*)
Radiation source: fine-focus sealed tube	*R*_int_ = 0.073
graphite	θ_max_ = 27.5°, θ_min_ = 1.1°
ω scans	*h* = −7→7
14228 measured reflections	*k* = −9→9
2011 independent reflections	*l* = −46→46

### Refinement

**Table:**

Refinement on *F*^2^	Primary atom site location: structure-invariant direct methods
Least-squares matrix: full	Secondary atom site location: difference Fourier map
*R*[*F*^2^ > 2σ(*F*^2^)] = 0.038	Hydrogen site location: inferred from neighbouring sites
*wR*(*F*^2^) = 0.117	H atoms treated by a mixture of independent and constrained refinement
*S* = 1.12	*w* = 1/[σ^2^(*F*_o_^2^) + (0.0674*P*)^2^] where *P* = (*F*_o_^2^ + 2*F*_c_^2^)/3
2011 reflections	(Δ/σ)_max_ = 0.001
213 parameters	Δρ_max_ = 0.37 e Å^−^^3^
1 restraint	Δρ_min_ = −0.33 e Å^−^^3^

### Fractional atomic coordinates and isotropic or equivalent isotropic displacement parameters (Å^2^)

**Table:**

	*x*	*y*	*z*	*U*_iso_*/*U*_eq_	
O1	0.4671 (3)	0.4022 (3)	0.89359 (5)	0.0161 (4)	
O2	0.1026 (4)	0.3085 (3)	0.88101 (5)	0.0237 (5)	
O3	0.5062 (3)	0.3882 (3)	0.78913 (5)	0.0166 (4)	
O4	0.1819 (4)	0.5944 (3)	0.74537 (5)	0.0204 (5)	
O5	0.4387 (4)	0.9627 (3)	0.80058 (6)	0.0220 (5)	
H5	0.541 (5)	0.999 (5)	0.7854 (8)	0.032 (10)*	
O6	0.6886 (3)	0.7118 (3)	0.84539 (5)	0.0164 (4)	
O7	0.5199 (4)	0.8762 (3)	0.89191 (6)	0.0251 (5)	
C1	0.1872 (5)	0.4312 (4)	0.94156 (7)	0.0152 (6)	
C2	0.3442 (5)	0.5291 (4)	0.96376 (8)	0.0177 (6)	
H2	0.4890	0.5684	0.9536	0.021*	
C3	0.2893 (6)	0.5693 (4)	1.00069 (8)	0.0208 (6)	
H3	0.3963	0.6364	1.0158	0.025*	
C4	0.0783 (6)	0.5115 (4)	1.01552 (8)	0.0207 (6)	
H4	0.0423	0.5364	1.0410	0.025*	
C5	−0.0805 (5)	0.4172 (4)	0.99317 (8)	0.0203 (6)	
H5A	−0.2256	0.3789	1.0034	0.024*	
C6	−0.0289 (5)	0.3787 (4)	0.95618 (8)	0.0183 (6)	
H6	−0.1395	0.3169	0.9408	0.022*	
C7	0.2420 (5)	0.3749 (4)	0.90243 (7)	0.0163 (6)	
C8	0.5387 (5)	0.3349 (4)	0.85715 (7)	0.0162 (6)	
H8A	0.4820	0.2058	0.8537	0.019*	
H8B	0.7108	0.3329	0.8558	0.019*	
C9	0.4444 (5)	0.4570 (4)	0.82602 (7)	0.0143 (6)	
C10	0.2631 (5)	0.3857 (4)	0.80053 (7)	0.0167 (6)	
H10	0.1942	0.2615	0.8067	0.020*	
C11	0.1072 (5)	0.5236 (4)	0.78182 (7)	0.0177 (6)	
H11	−0.0640	0.5067	0.7856	0.021*	
C12	0.1917 (5)	0.7166 (4)	0.77788 (7)	0.0173 (6)	
H12	0.0699	0.8160	0.7790	0.021*	
C13	0.4278 (6)	0.7684 (4)	0.79357 (7)	0.0170 (6)	
H13	0.5530	0.7316	0.7756	0.020*	
C14	0.4611 (5)	0.6656 (4)	0.83081 (7)	0.0141 (6)	
H14	0.3394	0.7078	0.8490	0.017*	
C15	0.6928 (6)	0.8232 (4)	0.87612 (7)	0.0183 (6)	
C16	0.9378 (6)	0.8664 (5)	0.88744 (9)	0.0274 (7)	
H16A	0.9366	0.9530	0.9087	0.041*	
H16B	1.0202	0.9235	0.8663	0.041*	
H16C	1.0169	0.7510	0.8947	0.041*	

### Atomic displacement parameters (Å^2^)

**Table:**

	*U*^11^	*U*^22^	*U*^33^	*U*^12^	*U*^13^	*U*^23^
O1	0.0167 (10)	0.0203 (11)	0.0112 (9)	−0.0012 (9)	0.0016 (8)	0.0001 (8)
O2	0.0184 (11)	0.0360 (13)	0.0167 (10)	−0.0048 (10)	−0.0022 (9)	−0.0016 (9)
O3	0.0193 (10)	0.0184 (10)	0.0120 (9)	0.0009 (9)	0.0021 (7)	−0.0031 (8)
O4	0.0252 (11)	0.0239 (11)	0.0122 (9)	−0.0022 (10)	−0.0031 (8)	0.0016 (8)
O5	0.0331 (13)	0.0125 (10)	0.0202 (10)	−0.0010 (10)	0.0035 (10)	0.0003 (8)
O6	0.0171 (10)	0.0177 (10)	0.0146 (9)	−0.0014 (9)	0.0000 (8)	−0.0013 (8)
O7	0.0250 (12)	0.0321 (13)	0.0182 (10)	0.0011 (11)	0.0004 (9)	−0.0079 (9)
C1	0.0159 (13)	0.0173 (14)	0.0124 (12)	0.0034 (12)	0.0009 (11)	0.0031 (10)
C2	0.0170 (14)	0.0161 (14)	0.0201 (13)	−0.0001 (12)	0.0029 (11)	0.0032 (11)
C3	0.0270 (16)	0.0167 (14)	0.0189 (14)	0.0011 (13)	−0.0008 (12)	−0.0014 (11)
C4	0.0282 (17)	0.0182 (15)	0.0158 (13)	0.0065 (13)	0.0048 (13)	0.0011 (11)
C5	0.0178 (15)	0.0201 (16)	0.0229 (15)	0.0016 (12)	0.0029 (12)	0.0044 (11)
C6	0.0152 (14)	0.0208 (14)	0.0190 (14)	0.0000 (13)	−0.0029 (11)	0.0042 (12)
C7	0.0168 (14)	0.0166 (14)	0.0155 (13)	−0.0006 (12)	−0.0017 (11)	0.0038 (11)
C8	0.0170 (14)	0.0183 (14)	0.0132 (12)	0.0021 (12)	0.0000 (11)	0.0006 (10)
C9	0.0160 (13)	0.0176 (14)	0.0094 (12)	0.0012 (12)	0.0015 (10)	−0.0011 (10)
C10	0.0178 (14)	0.0186 (14)	0.0137 (12)	−0.0004 (12)	0.0004 (11)	−0.0015 (11)
C11	0.0171 (14)	0.0237 (15)	0.0122 (12)	−0.0013 (12)	−0.0012 (11)	0.0001 (11)
C12	0.0207 (14)	0.0203 (14)	0.0110 (12)	0.0057 (13)	−0.0010 (11)	0.0009 (11)
C13	0.0232 (15)	0.0144 (14)	0.0133 (12)	−0.0008 (12)	−0.0003 (12)	−0.0007 (10)
C14	0.0131 (13)	0.0173 (14)	0.0120 (12)	0.0001 (11)	0.0017 (10)	−0.0005 (10)
C15	0.0236 (15)	0.0173 (15)	0.0141 (13)	0.0008 (13)	−0.0023 (12)	−0.0007 (11)
C16	0.0241 (16)	0.0281 (17)	0.0300 (16)	−0.0003 (15)	−0.0067 (14)	−0.0071 (14)

### Geometric parameters (Å, °)

**Table:**

O1—C7	1.345 (3)	C5—C6	1.382 (4)
O1—C8	1.448 (3)	C5—H5A	0.9500
O2—C7	1.206 (3)	C6—H6	0.9500
O3—C9	1.451 (3)	C8—C9	1.515 (4)
O3—C10	1.455 (3)	C8—H8A	0.9900
O4—C12	1.455 (3)	C8—H8B	0.9900
O4—C11	1.462 (3)	C9—C10	1.475 (4)
O5—C13	1.418 (3)	C9—C14	1.509 (4)
O5—H5	0.84 (3)	C10—C11	1.492 (4)
O6—C15	1.358 (3)	C10—H10	1.0000
O6—C14	1.445 (3)	C11—C12	1.474 (4)
O7—C15	1.204 (4)	C11—H11	1.0000
C1—C2	1.391 (4)	C12—C13	1.514 (4)
C1—C6	1.398 (4)	C12—H12	1.0000
C1—C7	1.488 (4)	C13—C14	1.532 (4)
C2—C3	1.386 (4)	C13—H13	1.0000
C2—H2	0.9500	C14—H14	1.0000
C3—C4	1.387 (4)	C15—C16	1.497 (4)
C3—H3	0.9500	C16—H16A	0.9800
C4—C5	1.388 (4)	C16—H16B	0.9800
C4—H4	0.9500	C16—H16C	0.9800
			
C7—O1—C8	115.8 (2)	O3—C10—C11	116.3 (2)
C9—O3—C10	61.00 (17)	C9—C10—C11	118.1 (3)
C12—O4—C11	60.70 (18)	O3—C10—H10	116.9
C13—O5—H5	103 (3)	C9—C10—H10	116.9
C15—O6—C14	116.2 (2)	C11—C10—H10	116.9
C2—C1—C6	119.9 (3)	O4—C11—C12	59.43 (17)
C2—C1—C7	122.3 (3)	O4—C11—C10	116.9 (2)
C6—C1—C7	117.7 (3)	C12—C11—C10	117.9 (3)
C3—C2—C1	120.0 (3)	O4—C11—H11	116.7
C3—C2—H2	120.0	C12—C11—H11	116.7
C1—C2—H2	120.0	C10—C11—H11	116.7
C2—C3—C4	120.0 (3)	O4—C12—C11	59.86 (18)
C2—C3—H3	120.0	O4—C12—C13	118.5 (2)
C4—C3—H3	120.0	C11—C12—C13	119.3 (2)
C3—C4—C5	120.1 (3)	O4—C12—H12	115.9
C3—C4—H4	120.0	C11—C12—H12	115.9
C5—C4—H4	120.0	C13—C12—H12	115.9
C6—C5—C4	120.4 (3)	O5—C13—C12	110.3 (3)
C6—C5—H5A	119.8	O5—C13—C14	108.3 (2)
C4—C5—H5A	119.8	C12—C13—C14	108.3 (2)
C5—C6—C1	119.6 (3)	O5—C13—H13	110.0
C5—C6—H6	120.2	C12—C13—H13	110.0
C1—C6—H6	120.2	C14—C13—H13	110.0
O2—C7—O1	123.2 (3)	O6—C14—C9	109.0 (2)
O2—C7—C1	124.2 (3)	O6—C14—C13	108.4 (2)
O1—C7—C1	112.6 (2)	C9—C14—C13	111.8 (2)
O1—C8—C9	111.4 (2)	O6—C14—H14	109.2
O1—C8—H8A	109.3	C9—C14—H14	109.2
C9—C8—H8A	109.3	C13—C14—H14	109.2
O1—C8—H8B	109.3	O7—C15—O6	123.3 (3)
C9—C8—H8B	109.3	O7—C15—C16	125.7 (3)
H8A—C8—H8B	108.0	O6—C15—C16	110.9 (3)
O3—C9—C10	59.64 (16)	C15—C16—H16A	109.5
O3—C9—C14	115.2 (2)	C15—C16—H16B	109.5
C10—C9—C14	117.3 (3)	H16A—C16—H16B	109.5
O3—C9—C8	112.4 (2)	C15—C16—H16C	109.5
C10—C9—C8	120.3 (3)	H16A—C16—H16C	109.5
C14—C9—C8	117.9 (2)	H16B—C16—H16C	109.5
O3—C10—C9	59.37 (17)		
			
C6—C1—C2—C3	2.1 (4)	O3—C10—C11—O4	23.0 (4)
C7—C1—C2—C3	−176.5 (3)	C9—C10—C11—O4	90.6 (3)
C1—C2—C3—C4	0.2 (4)	O3—C10—C11—C12	−44.9 (3)
C2—C3—C4—C5	−1.5 (4)	C9—C10—C11—C12	22.7 (4)
C3—C4—C5—C6	0.6 (4)	C11—O4—C12—C13	109.2 (3)
C4—C5—C6—C1	1.6 (4)	C10—C11—C12—O4	106.4 (3)
C2—C1—C6—C5	−2.9 (4)	O4—C11—C12—C13	−107.9 (3)
C7—C1—C6—C5	175.7 (3)	C10—C11—C12—C13	−1.5 (4)
C8—O1—C7—O2	−4.1 (4)	O4—C12—C13—O5	133.7 (2)
C8—O1—C7—C1	174.5 (2)	C11—C12—C13—O5	−156.8 (2)
C2—C1—C7—O2	−171.5 (3)	O4—C12—C13—C14	−107.9 (3)
C6—C1—C7—O2	9.9 (4)	C11—C12—C13—C14	−38.4 (3)
C2—C1—C7—O1	9.9 (4)	C15—O6—C14—C9	−128.9 (2)
C6—C1—C7—O1	−168.7 (2)	C15—O6—C14—C13	109.2 (2)
C7—O1—C8—C9	72.9 (3)	O3—C9—C14—O6	−93.0 (3)
C10—O3—C9—C14	−108.2 (3)	C10—C9—C14—O6	−160.3 (2)
C10—O3—C9—C8	113.0 (3)	C8—C9—C14—O6	43.4 (3)
O1—C8—C9—O3	−177.7 (2)	O3—C9—C14—C13	26.7 (4)
O1—C8—C9—C10	−110.8 (3)	C10—C9—C14—C13	−40.6 (4)
O1—C8—C9—C14	44.7 (4)	C8—C9—C14—C13	163.2 (2)
C9—O3—C10—C11	108.5 (3)	O5—C13—C14—O6	−61.7 (3)
C14—C9—C10—O3	104.6 (3)	C12—C13—C14—O6	178.7 (2)
C8—C9—C10—O3	−99.8 (3)	O5—C13—C14—C9	178.2 (3)
O3—C9—C10—C11	−105.5 (3)	C12—C13—C14—C9	58.6 (3)
C14—C9—C10—C11	−0.9 (4)	C14—O6—C15—O7	3.2 (4)
C8—C9—C10—C11	154.7 (3)	C14—O6—C15—C16	−177.8 (2)
C12—O4—C11—C10	−108.0 (3)		

### Hydrogen-bond geometry (Å, °)

**Table:**

*D*—H···*A*	*D*—H	H···*A*	*D*···*A*	*D*—H···*A*
O5—H5···O4^i^	0.84 (3)	2.05 (3)	2.887 (3)	172 (4)

Symmetry codes: (i) −*x*+1, *y*+1/2, −*z*+3/2.
